# Cognitive Changes in the Spinocerebellar Ataxias Due to Expanded Polyglutamine Tracts: A Survey of the Literature

**DOI:** 10.3390/brainsci7070083

**Published:** 2017-07-14

**Authors:** Evelyn Lindsay, Elsdon Storey

**Affiliations:** Department of Medicine (Neuroscience), Monash University (Alfred Hospital Campus), Commercial Road, Melbourne, VIC 3004, Australia; elsdon.storey@monash.edu

**Keywords:** spinocerebellar ataxias, dentatorubral pallidoluysian atrophy, expanded polyglutamine tracts, cognition, neuropsychological changes

## Abstract

The dominantly-inherited ataxias characterised by expanded polyglutamine tracts—spinocere bellar ataxias (SCAs) 1, 2, 3, 6, 7, 17, dentatorubral pallidoluysian atrophy (DRPLA) and, in part, SCA 8—have all been shown to result in various degrees of cognitive impairment. We survey the literature on the cognitive consequences of each disorder, attempting correlation with their published neuropathological, magnetic resonance imaging (MRI) and clinical features. We suggest several psychometric instruments for assessment of executive function, whose results are unlikely to be confounded by visual, articulatory or upper limb motor difficulties. Finally, and with acknowledgement of the inadequacies of the literature to date, we advance a tentative classification of these disorders into three groups, based on the reported severity of their cognitive impairments, and correlated with their neuropathological topography and MRI findings: group 1—SCAs 6 and 8—mild dysexecutive syndrome based on disruption of cerebello-cortical circuitry; group 2—SCAs 1, 2, 3, and 7—more extensive deficits based largely on disruption of striatocortical in addition to cerebello-cerebral circuitry; and group 3—SCA 17 and DRPLA—in which cognitive impairment severe enough to cause a dementia syndrome is a frequent feature.

## 1. Introduction

### 1.1. The Expanded Polyglutamine Tract Ataxias

Spinocerebellar ataxias (SCAs) types 1, 2, 3, 6, 7, 17 (named in order of discovery) and dentatorubral pallidoluysian atrophy (DRPLA) are dominantly-inherited expanded polyglutamine tract disorders that often or usually present with ataxia—reviewed in [1]. In addition, SCA 8 is also due, in part, to expression of an expanded polyglutamine tract [2]. Yet these disorders also differ in their pathological topography, and these differences underlie the differences seen clinically, radiologically and cognitively, at least at the group level.

### 1.2. The Potential Pathological Bases for Cognitive Impairment in the Polyglutamine Ataxias

#### 1.2.1. The Cerebellar Cognitive Affective Syndrome

The clinical features of many of the cerebellar ataxias arise from the degeneration of both cerebellar and extracerebellar structures, and it has been argued that the presence of cognitive dysfunction is due to extracerebellar compromise. However, the last three decades have seen an accumulating body of evidence for, and acceptance of, the proposition that the cerebellum itself plays a role in cognition. Anatomical investigations [3] and the use of anterograde and retrograde trans-synaptic tracing techniques in primates [4,5] provided foundational evidence for closed cortico-ponto-cerebello-thalamo-cortical loops, which link the prefrontal cortex—typically associated with executive function—with the phylogenetically-recent ventral and lateral macrogyric region of the cerebellar dentate nuclei and the associated posterolateral cerebellar cortex. Cerebellar output proceeds to the ventrolateral and medial dorsal nuclei of the thalamus, and thence to the prefrontal cortex [6,7]. Separate and non-overlapping closed loops also connect distinct cerebellar regions with cerebral motor regions. Diffusion tensor imaging (DTI) later verified the existence of prefrontal-pontine projections in the cortico-ponto-cerebellar portion of the loop in humans [8]. Further support was offered by functional connectivity magnetic resonance imaging (MRI), which demonstrated coupling of separate and specific areas of the cerebellum with association areas involved in cognitive functioning such as the prefrontal and parietal cortices, and the limbic system [9], see [10] for a review.

Based on bedside examination of 20 adults with localised cerebellar lesions, the seminal article by Schmahmann and Sherman [11] provided the framework for a constellation of nonmotor symptoms associated with cerebellar insult. Known as the ‘cerebellar cognitive affective syndrome’ (CCAS), it describes a range of impairments including executive dysfunction (deficits in planning, set shifting and abstract reasoning), language (semantic category fluency), spatial cognition (visual organisation and visual memory) associated with posterior cerebellar lobe lesions, and less well-characterised changes in affect and personality (blunting and disinhibition) associated with posterior vermis lesions. In contrast, lesions of the anterior cerebellum only produced motor impairment. The researchers ascribed the CCAS to disruption of neural circuitry linking different regions of the posterior cerebellar cortex with cerebral cortical association areas and paralimbic regions involved in higher order cognitive processing.

#### 1.2.2. Prefrontal Cortico-Striato-Thalamo-Cortical Loops

A number of spinocerebellar ataxias such as SCAs 2, 3 and 17 may also present with parkinsonism (vide infra), which suggests that the analogous cortico-striato-thalamo-cortical closed loops [12]—hereafter termed frontostriatal circuitry—may well be implicated in the genesis of cognitive dysfunctions in these disorders, in addition to motor impairment. The dorsolateral and orbitofrontal prefrontal-striatal circuits project from the basal ganglia to the prefrontal cortex via the ventroanterior and the dorsomedial regions of the thalamus [12]. Disruption in this circuitry presents as a ‘fronto-subcortical’ profile with a pattern of deficits such as impaired set shifting and spatial working memory [13,14].

#### 1.2.3. Cholinergic Projections from the Basal Forebrain Nuclei

The basal forebrain nuclei provide the major cholinergic input to neocortex, hippocampus and amygdala. Evidence of deficits in verbal and visual memory in ataxic patients—cognitive functions not typically associated with disruptions in the cerebro-cerebellar or frontostriatal systems—prompted the hypothesis that degeneration in the basal forebrain-cholinergic projection system may be contributory, as seen in patients with Alzheimer’s disease. Kish et al. [15] found marked cortical cholinergic denervation in the brains at postmortem of seven patients in a single pedigree with dominantly-inherited olivopontocerebellar atrophy (later classified as having SCA 1). Although finding substantial neuronal loss in the cholinergic basal forebrain nuclei of three patients with SCA 1, Rüb et al. [16] reported that these patients had shown no cognitive decline. Prominent degeneration of the cholinergic basal forebrain nuclei is also seen in SCA 2, but is less marked or absent in SCAs 3, 6, 7 and 17 [17].

#### 1.2.4. Direct Cortical Involvement

As will be outlined under individual disease entities below, it is apparent from radiological studies and a limited number of postmortems that some SCAs directly affect neurons of the association cortices themselves. Moreover, cortical neuronal loss can be underestimated without the benefit of stereological studies, which to the best of our knowledge have not been applied systematically across the polyglutamine ataxias.

### 1.3. Testing Executive Function in the Degenerative Ataxias—Pitfalls and Suggestions

#### 1.3.1. Potential Confounding Factors in Cognitive Studies of the Degenerative Ataxias

Studies of the cerebellum and cognition have encountered a number of confounding factors, not least of which is inconsistency between studies in test selection from the wide range of standard neuropsychological tests available. Not only does this lack of standardisation result in difficulties comparing study results and performing meta-analyses, but in some studies the choice of test(s) may not be sensitive to the nature of the deficits seen in the CCAS. In yet others, the tests chosen may require rapid verbal output, motor speed and accuracy, or rapid visual scanning, which may be regarded as non-cognitive test confounders. A further consideration when interpreting cognitive tests in ataxia patients is the potential cost of motor demands on attentional resources of those who ostensibly have adequate motor function for the task, as shown by Timmann and colleagues [18].

These non-cognitive test confounders potentially make it difficult to determine whether an observed cognitive deficit or dysfunction is merely secondary to the visual, speech and motor deficit(s) associated with that particular ataxia. As it is expected that cognitive dysfunction and these other deficits will progress together, it may also be difficult to establish an accurate temporal pattern of decline due to the CCAS on serial assessment of performance. There is also the issue of test choice appropriate to the deficits expected in the CCAS. As the core cognitive profile of the CCAS is frontal executive dysfunction, it is important to note that a commonly employed screening test for cognitive impairment or dementia is the Mini Mental State Examination (MMSE) [19]. Although used frequently in research and in the clinic, it is insensitive to executive function and is likely to overestimate cognitive status in ataxic patients [20]. The MMSE also omits a recognition component on testing recall, and the visuospatial item (pentagons) requires motor accuracy.

#### 1.3.2. Tests of Executive Function Suitable for Studies of Subjects with Degenerative Ataxias

Executive function tests that avoid the pitfalls of performance speed pressure, and do not place a premium on motor or visual speed and accuracy include the Wisconsin Card Sorting Test (WCST), Raven’s Progressive Matrices and the Zoo Map subtest from the Behavioural Assessment of the Dysexecutive Syndrome (BADS) battery. The Tower of London and Tower of Hanoi on the other hand, are examples of unsuitable tests as they are timed and require a degree of motor accuracy. Not all timed tests are unsuitable for use in ataxic subjects, however: some have a timed internal control component than can be used to account for visual scanning, articulatory and upper limb motor deficits. Examples include the Trail Making test and the Stroop test. Dysarthria is potentially a confounding factor for tests requiring verbal output such as the phonemic Controlled Oral Word Association Test (COWAT). However, it has been noted that the speed of articulation is not the rate-limiting factor on this measure [20].

#### 1.3.3. Are There Ideal Subjects for Cognitive Studies in Patients with Cerebellar Disorders?

Finally, investigation of the role of the cerebellum in cognition demands the availability of patients for assessment who have damage restricted to the cerebellum, with no extracerebellar involvement. Patients who have had a cerebellar stroke are sought for carefully controlled studies, and have revealed a range of neuropsychological deficits and neuropsychiatric disturbances for reviews see [21,22]. However, strokes, by their focal nature, may not include cerebellar regions critical for cognition. They also tend to recover, at least partially — reviewed in [21]. Both problems would potentially be overcome by studying those with degenerative cerebellar disease, but patients who have widespread and diffuse pathology with complete sparing of all other central nervous system structures are essentially non-existent. While considered to be relatively ‘pure’ cerebellar ataxias, SCA 6 and SCA 8 both have mild extracerebellar pathological changes, as illustrated by common non-ataxic clinical features such as pyramidal signs. However, the relatively ‘pure’ SCAs such as SCAs 6 and 8 do represent the most appropriate means of studying the CCAS in degenerative ataxias.

### 1.4. Scope of This Literature Survey

This article surveys the cognitive changes reported in the polyglutamine ataxias, in the light of their pathological, radiological and clinical features.

## 2. Spinocerebellar Ataxias

### 2.1. SCA 1

SCA 1 is due to a cytosine-adenine-guanidine (CAG)_n_ repeat expansion in the ataxin-1 (*ATXN1)* gene [23], resulting in an expanded polyglutamine (Q_n_) tract of between 39 and 91 residues. It tends to show anticipation—earlier onset in successive generations on the basis of an intergenerational increase in (CAG)_n_ size—with a wide range of onset age (mean in the 4th decade), and a total disease duration of about 20 years (reviewed in [24]). Due to founder effects, it is the commonest SCA in Russia, Poland, northern Italy and South Africa, and is a close second (to SCA 6) in Australia.

SCA 1 causes marked atrophy of the brainstem, as well as of cerebellar cortex (Purkinje cells), deep cerebellar nuclei, spinocerebellar tract, red nucleus, ventral posterolateral nucleus of the thalamus and the Betz cells of the motor cortex [17]. Of particular relevance to cognition, there is also marked neuronal loss in the cholinergic forebrain nuclei (bands of Broca, basal nucleus of Meynert) and the mediodorsal nucleus of the thalamus, with loss of volume of the prefrontal and frontal cortex [25] and severe loss of neurons in the cerebral cortex [17]. The pallidum, ventral anterior nucleus of the thalamus and substantia nigra pars compacta may also be affected to a lesser extent [17].

Volumetric MRI studies reviewed by [26,27] reveal atrophy of the cerebellar vermis and hemispheres as well as the middle cerebellar peduncle and brainstem (especially the basis pontis, but also the medulla), and the cervical spinal cord in an olivopontocerebellar atrophy-type pattern. The basal ganglia and cerebral hemispheres are reported to be unaffected radiologically (ibid) notwithstanding the neuropathological findings listed above.

The clinical features of SCA 1 have been reviewed [24]. In addition to ataxia, about 50% of patients have upper motor neuron signs (sometimes with prominent spasticity), although this may be disguised if neuropathy intervenes. Nystagmus is evident only in a minority (~25%), although moderately slow, hypermetric saccades are common (~50%), and ophthalmoplegia can develop in about 30%. Chorea or dystonia may be evident in a minority (~15%).

Relatively mild cognitive changes have been reported variously in 5–25% in advanced stages of the disease [28] and in 20–30% of cases [24]. The following studies were performed with a range of measures, only some of which were appropriate, as discussed in Section 1.3. Possible confounding factors related to assessment tools are discussed in detail for each study where information was available. Using cognitive tasks without a limiting motor component (e.g., WCST, verbal auditory memory tests, Raven’s Progressive Matrices and Wechsler Adult Intelligence Scale-Revised (WAIS-R) subtests), early neuropsychological assessment of a large North American kindred (the Schut pedigree) with SCA 1 found 11 of 14 family members with mildly reduced verbal and non-verbal intellectual ability, memory and executive function compared with controls, on assessment tasks without a limiting motor component [29]. As a methodological confound was identified in the demographic matching of controls, who had significantly higher intelligence quotients (IQs) that likely exaggerated the extensive relative cognitive deficits in the patient group, a subsequent study neuropsychologically assessed 14 patients from unrelated SCA 1 pedigrees and 11 appropriately matched controls [30]. Controlling for age (<65 years) and using tests to minimise the effects of limb ataxia and dysarthria, results revealed prominent executive dysfunction, characterised by perseverations and set shifting difficulties, and reduced verbal memory. It was noted that no recognition component was included in the memory task, thus a simple retrieval deficit could not be excluded as a possible cause of the reduced memory. Attention, visuospatial processing and visual memory were spared. General intellectual impairment as assessed by the MMSE, the commonly-used brief screening tool for dementia, was observed in only some of the participants, suggesting that it was not a typical feature in the early stage of the SCA 1 phenotype.

Overall, other studies employing both the MMSE and appropriate neuropsychological tests have reported that patients with SCA 1 exhibit mild cognitive decline which rarely progresses to a dementia [31,32,33,34,35]. Executive dysfunction is the most commonly reported cognitive feature in SCA 1 patients [29,30,31,34,35], and is more prominent and with higher error rates than observed in patients with SCA 2 and SCA 3 [31]. While most studies used the WCST as a measure of executive function, Klinke et al. [34] administered the timed Tower of Hanoi test to their SCA 1, 2, 3 and 6 patients, reporting that none of the SCA patients were in the impaired range. However, on other tests of executive function and compared to age-, gender-, and IQ-matched controls, 50% of their SCA 1 patients showed impaired phonemic verbal fluency and made a greater number of errors on an inverse choice reaction task when compared to test norms following post hoc analyses. The study controlled for ataxia severity and fine motor coordination, as measured by performance on the Purdue Pegboard, a timed test of placing pegs in small holes with either, or both, hands.

Verbal memory has also been examined in SCA 1 patients [29,30,31,34,35,36,37] with variable results. It is unclear in early studies if measures used included a recognition component, as noted by Bürk et al. [30]. Administering both recall and recognition tasks, three studies [34,35,36,37] found relatively intact verbal memory in SCA 1 patients in contrast to previously documented distinct verbal memory deficits in SCA 1 [29,30,31]. This inconsistency was attributed to differences in the memory tests used [34,35,36] and suggested to the researchers that temporal lobe function is relatively preserved in SCA 1 patients [34]. Of interest, Orsi et al. [37] reported a similar level of verbal learning for SCA 1, 2, 3 and 6 patients to controls, although they noted that all patients demonstrated inefficiency in consolidation across trials and in delayed recall. In contrast, performance on a recognition task was comparable to controls. This apparent deficit in the memory retrieval process was considered more typical of compromised cerebellar and prefrontal regions than mesial temporal lobe structures.

On social cognition tests (e.g., attributing five basic emotions of happiness, sadness, fear, anger and embarrassment to short story characters), SCA 1 patients showed no impairment relative to patients with SCA 2 or SCA 3 [36], although no control group was included for comparison. Depression has been observed to be common, at both early and late stages of the disease [37] and is independent of ataxia severity [29,38], suggesting that other factors must be considered, such as the emotional response to nonmotor changes (i.e., cognition) rather than simply motor disability, as well as possibly being an inherent feature of the pathological changes in brain circuitry, as postulated for the CCAS. In sum and notwithstanding confounding factors and impairment heterogeneity among SCA 1 patients, these studies have shown executive dysfunction, together with some inefficiency in memory, providing some support for disruption in the cerebro-ponto-cerebello-thalamo-cortical network, and extending to corticostriatal circuitry and/or possible cortical or cortical projection involvement from the basal forebrain.

### 2.2. SCA 2

SCA 2, concisely reviewed by Aubuger [39], is due to a (CAG)_n_ repeat in the *ATXN2* gene, coding for ataxin 2 [40,41,42]. The mutation results in an expanded polyglutamine tract, ranging from 33 to >200 residues. Like SCA 1, it tends to show anticipation. There is a wide range of onset ages, from 2 to over 70 years, but is typically first manifest in the 3rd to 5th decade. The duration of disease is also highly variable, ranging from 6 to 50 years. It is the commonest SCA in India, Spain, Cuba, southern Italy and Korea.

The topography of neuropathological involvement is very similar to that of SCA 1, except for greater involvement of the medial septal nucleus of the forebrain, the pallidum, substantia nigra pars compacta and the ventral tegmental area, reticular, ventral anterior and ventrolateral nuclei of the thalamus and the pulvinar, and less involvement of the deep cerebellar nuclei and the spinocerebellar tracts [17,25]. There may be considerable cerebral cortical neuronal loss [17], with atrophy of the frontal lobe [25].

MRI findings in SCA 2 are similar to those in SCA 1 [26,43], although the brainstem atrophy tends to be even more severe, and may result in the “hot cross bun” sign typically seen in MSA_C_ (multiple system atrophy of cerebellar type) in 25% of patients [44]. Of particular relevance to this review, there may also be atrophy of the cerebral cortex in advanced disease [45,46].

Characteristic clinical findings include extremely slow saccades (without nystagmus), initial hyperreflexia with supervening hypo/areflexia, postural tremor, and sometimes myoclonus [39]. Cognitive impairments may be more pronounced in some pedigrees, e.g., [20] than the usual mild dysexecutive syndrome common to the degenerative ataxias, but overall impairment is generally considered to be uncommon [47]. SCA 2 may also present with late-onset L-dopa-responsive parkinsonism, especially in those of East Asian origin see [39].

The frequency of cognitive deficits has been estimated to be 5% to 19% in SCA 2 patient groups overall [28,48]. Storey et al. [20] pointed out that early studies only variably undertake systematic assessment of cognitive function, or often fail to assess or report cognitive impairment or possible dementia. Using assessment measures to control for motor and visual impairment as outlined in Section 1.3, Storey et al. [20] found seven of eight patients from one pedigree with SCA 2 demonstrated impairment on tests of frontal lobe function (verbal fluency, Stroop, and WCST). A qualitatively abnormal result on Luria’s fist/edge/palm of motor regulation was also observed and was not considered by its nature to be compromised by cerebellar motor dysfunction. With clear evidence of frontal-executive dysfunction, comment was made that the disparity between the results of these tests and MMSE scores (25 and above) highlighted the insensitivity of the MMSE to frontal lobe dysfunction and the consequent possibility of missing significant cognitive impairment with this instrument. A study administering a cognitive battery which included tests of social cognition, found that SCA 2 patients had greater cognitive impairment than patients with SCA 1 and SCA 7, specifically in frontal executive function, attention and social cognition [36].

A small number of earlier studies implemented some assessment tools appropriate to the patient group although certain results remain inconclusive due to test confounds. A study of the clinical features of SCA 2 by Gambardella et al. [49] utilising generally relevant neuropsychological assessment tools with 11 patients from three unrelated families from southern Italy, found early impairment of conceptual reasoning ability, as measured by the WCST, in five individuals. The study also included a computerised eye movement method to record saccadic eye movements and smooth pursuit eye movements, with results showing that slow saccades occur from early in the SCA 2 disease process. Of note, poor performance on the WCST was strongly associated with decreased saccadic velocity, leading to speculation by the researchers of degeneration in cortical as well as cerebellar regions or, indeed, as a consequence of disruption in cortical-subcortical circuitry. Performances on memory tasks, language function, and on a depression screen were intact relative to controls. Although the assessment method for determining dementia was not described, an early large study examining SCA 2 pedigrees reported dominant dementia and rapid disease course in a single SCA 2 family [50]. Concordant with this, Bürk et al. [48], using standardised cognitive tests, found 25% of their 17 SCA 2 patients showed evidence of dementia (<23/30 on the MMSE) while patients without dementia exhibited impaired memory (characteristics of test performance were not described and a recognition trial was not performed) and executive function (on the WCST and verbal fluency tests). The presence of dementia was associated with disease duration but not with (CAG)_n_ repeat size or with age of onset. Further, implementing a comprehensive test battery in 18 SCA 2 patients from eight families, Le Pira et al. [51] confirmed low cognitive status, which was characterised by reduced verbal fluency, retrieval-based verbal memory impairment and reduced attention.

Compared with other autosomal dominant cerebellar ataxias, SCA 2 has been most commonly associated with dementia. Sokolovsky et al. [52] found the cognitive abilities of SCA 2 patients to be more deficient than SCA 1, SCA 6 and SCA 7 patients, particularly in attention, non-verbal intelligence and executive functioning on tests generally appropriate for patients with motor impairment. However, a longitudinal investigation over seven years of cognitive and disease progression revealed SCA 2 and SCA 6 patients declined *less* than SCA 1 and SCA 3 patients on cognitive and cerebellar examinations, the latter quantified by the Scale for the Rating and Assessment of Ataxia (SARA) [52]. The reasons for, and basis of, the prominent differences in cognitive impairment between SCA 2 pedigrees is unclear.

Viewed as a “frontal-subcortical” profile and supportive of the disconnection hypothesis, neuronal loss in SCA 2 is not restricted to the cerebellum and involves, to greater or lesser degree, the pallidoluysian system and substantia nigra. In keeping with this, the cognitive profile of SCA 2 patients, especially those with greater cognitive impairment, does not support a purely cerebellar cause [20].

### 2.3. SCA 3

SCA 3, also known as Machado–Joseph disease (MJD), has been well-reviewed by Paulson [53]. It is due to a (CAG)_n_ repeat in the *ATXN3* gene, coding for ataxin 3 [54]. The mutation results in an expanded polyglutamine tract, ranging in size from 51 to 89 residues. As with SCAs 1 and 2, it tends to show anticipation. SCA 3 is the commonest dominant SCA world-wide, and specifically in the United States, Germany, France, Portugal, Brazil, Japan and China. The onset age is typically in the 3rd to 5th decades, but, as with SCAs 1 and 2, varies widely (from 5 years to 70 years in the case of SCA 3).

Pathologically, SCA 3 affects the deep cerebellar nuclei (especially the dentate and fastigial) and the red nucleus prominently, with the cerebellar cortex and inferior olive less severely affected than in SCAs 1 and 2 [17,25]. The pallidum, subthalamic nucleus, substantia nigra pars compacta and ventral tegmental area, ventro-anterior nucleus of the thalamus, spinocerebellar tracts and pedunculopontine nucleus are also severely affected. The cholinergic nuclei of the basal forebrain are spared, and the cerebral cortex is typically not macroscopically involved [25] although there is severe neuronal loss [17].

Reports of neuroradiological features of SCA 3 have varied considerably [43], but an olivopontocerebellar atrophy (OPCA)-type pattern, albeit usually less severe than that seen in SCAs 1 and 2, is generally seen. Reflecting this, the “hot cross bun” sign is uncommon in SCA 3, being seen in about 1% of cases [44].

The clinical heterogeneity of SCA 3 in part reflects the heterogeneity of age of onset (and therefore of the size of the (CAG)_n_ expansion) [53]. Three phenotypes were originally recognised: type I, with spasticity, rigidity and bradykinesia without prominent ataxia—typically of teenage or young adult onset; type II (the commonest), with ataxia and pyramidal signs—typically of young to mid-adult onset; and type III, with ataxia and peripheral neuropathy/amyotrophy—usually of onset in middle to late middle age [53]. A further phenotype (type IV) was later proposed, to cover those with a parkinsonian (and sometimes L-dopa-responsive) phenotype [53]. Unlike SCAs 1 and 2, nystagmus is seen in the majority, although ophthalmoplegia tends to supervene later.

Cognitive impairment in SCA 3 has generally been considered uncommon [47] with normal intellect reported across a number of clinical studies [55,56,57]. The first study to employ neuropsychological methodology with SCA 3 patients found subtle deficits in the verbal memory of affected family members compared with those at risk, and on a visual memory task in both groups [58]. The researchers attributed the visual memory performance to a defective perceptual ability due to impairment of visual scanning. Questioning the interpretation of this deficit, Maruff et al. [13] sought to clarify the cognitive profile in six indigenous Australian genetically-confirmed SCA 3 patients using the Cambridge Neuropsychological Test Automated Battery (CANTAB). The CANTAB is a touch screen neuropsychological testing system which has been standardised for normal participants and extensively used for cognitive assessment in a number of movement and neurodegenerative disorders. Consistent with previous studies, results indicated no global cognitive impairment or dementia, and visual memory was found to be intact. Conversely, and independent of motor function, specific deficits were evident in visual attention characterised by slowed processing of visual information with high task demand and difficulty with shifting attention, features suggestive of fronto-subcortical disruption. Using an appropriate neuropsychological test battery for movement disorders in six SCA 3 patients with no evidence of dementia or global cognitive impairment, Zawacki et al. [59] found clear impairments in specific executive abilities (phonemic fluency using the COWAT, timed attentional tasks, cognitive flexibility using both the Stroop test and WCST). Milder impairments were reported in attention to detail and cognitive processing speed on an oral version of the Symbol Digit Modalities Test. Depression was assessed to be in the moderate-to-severe range in four patients and apathy was observed. More extensive impairment was noted by Kawai et al. [60] in 16 genetically confirmed patients with SCA 3, who exhibited impairments in verbal and visual memory, and in verbal fluency, as well as in visuospatial and constructional function on untimed tests: deficits which were unrelated to CAG repeat length. In a large sample group of 38 patients with SCA 3, Brago-Neto et al. [61] reported a specific deficit in phonemic fluency (but not semantic fluency), and impaired processing speed and visuospatial attention compared with controls. Finding no impairment on memory recall and recognition tests, in contrast to other studies of SCA 3 patients, the researchers commented on the variety of memory tests used across studies. Depression was also a feature of this patient group. Prospectively examining 11 SCA 3 patients with a disease duration of 5.1 ± 2.8 years over a mean of three and a half years, Roeske et al. [62] reported cognitive deterioration from baseline in tasks initially performed at equivalent levels to age- and gender-matched controls, comprising verbal learning and memory, and figural memory. Mood remained within normal levels on a depression scale.

Using cognitive tests requiring minimal motor activity, Lopes et al. [63] identified mild deficits in episodic verbal memory and working memory in 24 SCA 3 patients (eight patients with significant depression were excluded), with additional mild reduction in a nonverbal abstract reasoning when compared to controls. These deficits were found not to be associated with (CAG)_n_ expansion size, disease duration or ataxia severity. Garrard et al. [64] found that memory impairment and executive dysfunction were present in both SCA 3 and SCA 6 patient groups (the latter with a “purer” cerebellar syndrome), although notably more pronounced in SCA 3.

Social cognition, as measured with a Theory of Mind (ToM) test, was found to be more impaired in SCA 3 (six) than in SCA 6 (nine) patients, although the deficit was restricted to physical rather than mental or emotional attributions, or judgements of social behaviour [64]. Although not conducted in SCA 1, social cognition tests have indicated impairment in SCA 2 patients and a more subtle effect in SCA 3 patients [36,64].

Overall, and contrary to the view that cognitive difficulties are rare in SCA 3, there is good evidence of cognitive dysfunction with a cognitive profile of SCA 3 that is considered reflective of cerebro-cortical dysfunction due to disruption in the cerebrocerebellar system and cortico-striatal-thalamocortical circuitry [33].

While frontal executive dysfunction and attention deficits appear to be common in SCA 1, SCA 2 and SCA 3, deficits in visual attention and processing speed have been reported more often in SCA 3. Deficient performances on memory tasks have been observed in SCA 1, SCA 2 and SCA 3 with memory reported to be more impaired in SCA 1. However, it has been noted that a range of different memory tasks have been used across studies which may place different demands on working memory, language processes and executive function [61]. Also, it is often uncertain whether a recognition task was used to identify a retrieval-based, and thus more subcortical, deficit [31]. Affective disturbances have also been noted and appear to be more common in SCA 3 patients than in SCA 1 or SCA 2 patients. Depression and anxiety symptoms and apathy were reported in a number of the SCA 3 studies cited above [59,61,63].

### 2.4. SCA 6

SCA 6 is due to a short (*n* = 20–33), intergenerationally stable (CAG)_n_ expansion in the *CACNA1A* encoded subunit of the PQ type calcium channel [65], although it is not clear that altered channel function is the pathomechanism of neuronal damage. SCA 6 is uncommon (about 1–2% of dominant SCAs) in France, Spain, Italy, Poland and South Africa [66], but >3% in other regions tested—notably about 25% in the Netherlands and Japan. The average age of onset is in the 5th to 6th decade—distinctly later than that of the other disorders reviewed here. It may sometimes be non-penetrant, and does not shorten life [66]. It is allelic with episodic ataxia type 2 (and familial hemiplegic migraine type 1), and may sometimes display episodic attacks of moderate to severe ataxia in the early stages of the disease [67].

The major pathological impact of SCA 6 is on the Purkinje cells of the cerebellar cortex and the giant pyramidal Betz cells of the motor cortex, although milder and variable neuronal loss is seen in the deep cerebellar nuclei, inferior olive, red nucleus, substantia nigra pars compacta, and the remainder of the cerebral cortex [17,25]. There is no macroscopic supratentorial atrophy. Radiologically, SCA 6 typically results in pure cerebellar cortical atrophy, particularly prominent superiorly [26,43].

Clinically, in addition to the usual features of ataxia in the SCAs, vertical nystagmus is seen in 65–83% of patients with SCA 6, but in <10% in other SCAs [68]. Hyperreflexia with extensor plantars is seen in up to half, and frank spasticity may occasionally be present.

As the pathological changes in SCA 6 are mostly restricted to the cerebellum, with selective loss of cerebellar Purkinje cells, researchers have sought to investigate the contribution of the cerebellum to higher order cognitive functions in this patient group. Initial studies [69,70,71] reported no evidence of intellectual impairment or dementia based on clinical examination, and the first systematic testing of cognition with a battery of neuropsychological tests without notable motor limitations on 12 patients with SCA 6 found no significant impairment of attention, working memory, verbal or visuospatial memory, or executive function [72]. Although not statistically significant, slightly lower performances than controls were in fact observed on tests of verbal memory, semantic fluency and executive function [28,71]. Suenaga et al. [73] used tests with minimal or absent reliance on motor function to compare 18 SCA 6 patients to controls, with results showing marked impairment on visuospatial memory, and phonemic and semantic fluency tasks. While not statistically significant after correction, the patients also performed worse than controls on a response inhibition test. There was no difference between groups on tests of attention and working memory. With measures sensitive to subtle cognitive deficits and avoiding visuomotor confounds, Garrard et al. [64] also found relative sparing of attentional functions in SCA 6 patients. Verbal immediate recall was noted to be impaired in two of nine patients. On executive function tests, SCA 6 patients showed deficits only on the Hayling Sentence Completion Task, a measure considered suitable for people with visual perceptual or motor problems which entails response initiation and response inhibition in verbally completing sentences with a nonsense word or suppressing sensible word endings. There was selective impairment on a social cognition task (ToM), and patients were significantly impaired on a more difficult response inhibition task than used previously. Administering a broad neuropsychological battery to 27 patients with SCA 6, Cooper et al. [74] reported impairments at the group level specifically related to tests of executive function (verbal and non-verbal reasoning, response inhibition and cognitive flexibility). Phonemic and category fluency, and category switching were impaired in a subset of patients. Intellectual functioning and memory were intact. Although some patients were unable to complete tests that involved a motor response due to their severe upper limb ataxia, and motor impediment may have contributed to the lower IQ on the WAIS-III Performance Scale, it was considered that the results supported a role for the cerebellum in executive dysfunction characterised by the CCAS, albeit in a subtle form due possibly to the superior-anterior to posterior-inferior progression of this largely cerebellar disease. Klinke et al. [34] reported that SCA 6 patients were significantly, although mildly, impaired in only two areas: attention (symbol counting) and executive function (errors on an inverse choice reaction task). Given the tasks involved motor function and the results of these patients were related to motor impairment and/or ataxia severity, poor performances may be explained by motor problems during the task or possibly attributed to the cerebellar role in cognitive processes with deterioration concurrent with motor impairment.

While none of the 32 patients with SCAs 1, 2, 3 and 6 in the study of Klinke et al. [34] produced scores of severe depression on the Beck Depression Inventory, mildly depressed mood was indicated in six of eight SCA 6 patients, three of six SCA 1 patients, and to a much lesser degree in SCA 2 and SCA 3 patients. In contrast to SCA types 1, 2 and 3 patients with extracerebellar pathology, who had cognitive profiles characterised by poor frontal attentional and executive deficits, the relatively mild attention and executive dysfunction in SCA 6 patients presumably reflected predominantly cerebellar compromise. Performing univariate logistic regression analyses, Schmitz-Hübsch et al. [75] identified only age of onset and disease duration to be associated with non-ataxia symptoms. This raised the possibility that, because of the later age of onset of SCA 6, certain non-ataxia symptoms may be the result of aging rather than the disease itself.

### 2.5. SCA 7

SCA 7 is due to a (CAG)_n_ repeat in *ATXN7*, coding for ataxin 7 [76], and results in an expanded polyglutamine tract. Perhaps due to the lack of stabilising non-CAG intrusions into the (CAG)_n_ repeat sequence, the expanded *ATXN7* allele (with 36–460 CAG repeats) is highly intergenerationally unstable, and may show extreme anticipation (e.g., a child manifesting before his/her father). As expected therefore, and as for SCAs 1, 2 and 3, the onset age ranges from infancy to old age. As with SCA 3, there is age/repeat size variation in clinical presentation (vide infra). SCA 7 is uncommon world-wide (usually about 2% of dominant SCA pedigrees), except in Scandanavia, the Netherlands and Japan, where frequencies of 10–15% have been found. SCA 7 has been reviewed in some detail by Martin [77].

Neuropathologically, SCA 7 is unique in displaying damage to the retinal pigment epithelium at the macula, particularly in patients with an age of onset <50 years, in whom it may be the presenting feature. Otherwise, it affects cerebellar Purkinje cells and the deep cerebellar nuclei (especially the fastigial), with less involvement of the red nucleus and inferior olive [17]. The pallidum, substantia nigra pars compacta, and ventro-anterior nucleus of the thalamus are mildly affected, and the cholinergic nuclei of the basal forebrain are spared, although the medial dorsal nucleus of the thalamus (linked to the prefrontal cortex) is severely impacted.

Radiologically, the pattern of atrophy can again be characterised as pontocerebellar, with one group reporting that pontine atrophy actually precedes cerebellar atrophy and is more severe than in SCA 3 [78], although the former point is contentious [77]. Alcauter et al. [79] found widespread cerebral cortical atrophy in a voxel-based morphometry study.

Clinically, SCA 7 tends to present with visual loss in those with high repeat numbers (≥60) and concomitant onset <50 years, while those with smaller repeat numbers and onset >50 years tend to present with ataxia [77]. Slow saccades, ultimately leading to ophthalmoplegia, pyramidal features and bulbar amyotrophy may also be evident.

Despite early reports by Benton et al. [80] of dementia in some numbers of a Saudi Arabian family (the RASCA kindred) presenting with progressive psychosis, auditory hallucinations, delusions, and behavioural disturbances, and perhaps because of its relative rarity, there is a dearth of studies examining and reporting on cognitive changes and psychiatric features in patients with SCA 7 [77]. Horton et al. [81] reported cognitive deficits identified during routine clinical care of 16 SCA 7 patients followed over 27 years, which included difficulties with multitasking, impaired phonemic fluency and reduced free recall. The means by which these deficits were identified were not described. The first known study to systematically assess cognition in SCA 7 compared the cognitive profiles of two SCA 1, three SCA 2, and three SCA 7 patients (and patients with SCA 3 and SCA 6 from a previous study using identical methodology [64]) [36]. Selective deficits with a cognitive profile similar to SCA 1 and SCA 6 were reported, primarily in executive function (phonemic fluency, the Hayling test, and response inhibition on the Stroop test), possibly reflecting early fronto-ponto-cerebellar degeneration. Impaired attention was noted in some patients although this was absent in the SCA 7 group. Memory impairments were observed in SCA 3, SCA 6 and SCA 7, while one SCA 7 patient who exhibited visual memory loss also had the longest disease duration which is usually characterised by visual loss [52]. Perceptual function and calculation ability were preserved in all groups. Of interest is the inclusion of social cognition tests in these studies, which demonstrated impairments in SCA 2 and to a lesser extent in SCA 7, on a task attributing five basic emotions to characters in short stories. Patients with SCA 1, SCA 3 and SCA 6 were unimpaired on this task. A ToM task revealed normal performances for SCA 2 and SCA 7 patients, and impairments in SCA 3, SCA 6 and one patient with SCA 1 [36]. While there is some emerging evidence of cognitive deficits and their nature in SCA 7 patients, given the very limited number of studies reporting on cognitive abilities based on appropriate assessment measures in this patient group, together with small sample size, results so far must be treated as preliminary with future studies needing to consider age of disease onset and vision impairment.

### 2.6. SCA 8

SCA 8 [82] appears to have a unique pathomechanism: a (CAG)_n_ repeat expansion in the *ATXN8* gene is translated to an almost purely polyglutamine protein, while the opposite strand (*ATXNOS*), with its complementary CTG repeats, may result in RNA-mediated toxicity [83]. It is unique in that there is a (CAG/CTG)_n_ pathogenic “window” of about 80–250 repeats, the expansion is markedly non-penetrant within this range, and in some pedigrees there is a bias towards transmission from females due to massive contractions of the repeat in sperm [84]. (Note that transmission from *males* tends to result in *greater* expansions in SCAs 1, 2, 3, 7 and DRPLA.) SCA 8 is particularly common in Finland, where it is responsible for 18% of dominant ataxia pedigrees, but it is not frequent elsewhere [85]. Its age of onset is varied—from 13 to 65 years in a recent review [84] —and progression is slow (e.g., 20 years to progress to walking aids).

Neuropathologically, neuronal loss is reported to affect Purkinje cells (with moderate to severe loss), and cerebellar granule cells, inferior olivary neurons and the substantia nigra (to a lesser extent) [84].

MRI shows prominent pan-cerebellar atrophy; the brainstem and cerebral hemispheres are spared—reviewed in [26].

The clinical presentation is of ataxia, with nystagmus in about ^2^/_3_. Mild pyramidal features are common and low amplitude distal myoclonus may be seen [84].

A number of studies reporting the clinical features of SCA 8 have briefly described cognitive impairment and psychiatric presentations although only a limited number have used neuropsychological measures. Memory impairment was noted on neurological examination of two patients (with repeat sizes of 132 and 152) and mental retardation in one young girl from a group of seven patients from five unrelated families [86]. Although not formally neuropsychologically assessed, six of 15 Finnish mutation carriers with large repeats exhibited mild and unspecified cognitive difficulties, two of whom also had, separately, borderline personality disorder and bipolar disorder. Two other patients without obvious cognitive impairment had been treated for depression [87]. Stone et al. [88] described a mother and son with SCA 8 expansion and mild cerebellar ataxia who were administered a neuropsychological battery. The son (aged 46 years) presented with a three-year history of mild progressive gait ataxia, impairment in manual dexterity and dysarthric and dysprosodic speech with slowed repetitive tongue movements. On testing and compared to age-matched controls, he demonstrated executive dysfunction, more impaired visual memory than verbal memory, and intact language. Executive function testing was appropriate in relation to using the WCST, and the Stroop test and a phonemic fluency task were also included. Visuospatial reasoning and timed visual scanning were both impaired although the tests rely on visuomotor and verbal processing. The MMSE score was pristine. Perseveration was observed, as well as a lack of concern for poor performance, and his wife reported a marked change in personality with labile mood and aggressive outbursts. His mother (75 years) exhibited inappropriate affect with neurological symptoms reported to be dysarthria and difficulty walking which she attributed to arthritis. Tests showed poor figure reproduction on the MMSE (with a total score of 29/30 within the normal range), reduced phonemic and semantic fluency, and impaired performance on a frontal executive motor test (the Luria three-step test). In a later study by these researchers, six unrelated patients with SCA 8 expansions causing ataxia were psychometrically tested with the MMSE and Addenbrooke’s Cognitive Examination (ACE) tool, both of which are commonly used as dementia screens, but are relatively insensitive to subtle cognitive changes and executive dysfunction—especially the MMSE. No evidence of dementia was found. However, psychiatric examination revealed a range of morbidities including past major depression and panic disorder occurring soon after ataxia onset, and obsessive-compulsive disorder following onset of neurological symptoms [89]. Lilja et al. [90] reported cognitive impairment in 10 SCA 8 patients who underwent comprehensive neuropsychological testing. To account for motor dysfunction, timed and untimed measures of many of the tests were implemented, and purely cognitive measures were used. Attention and information processing speed were assessed using tasks with similar motor and perceptual measures although with different cognitive requirements. Deficits in attention, information processing, concept formation reasoning, verbal production (as on verbal fluency tasks) and executive function were primarily identified, while visuoperceptual and constructional abilities, and most memory functions, were intact. As in the previous studies, the MMSE was noted to be inappropriate for evaluation of SCA 8 due to its insensitivity.

Although further evidence would be expected to be forthcoming, these studies identified areas of cognitive vulnerability in SCA 8 patients, most notably in attention and executive function. Affective changes and psychiatric features were also observed. Taken together, these results are so far consistent with features of the CCAS subserved by disturbance in the cerebro-cortical circuitry.

### 2.7. SCA 17

SCA 17 results from a (CAG) repeat expansion of between 41 and 63 in the gene encoding TATA Box Binding Protein (TBP) [91]. Unlike other relatively large (CAG) repeats, there is little intergenerational instability, probably due to (CAA) insertions into the repeat sequence [92]. It may be non-penetrant in the range from 41 to 48 repeats. SCA 17 is found world-wide, but is relatively rare everywhere e.g., see [85]. The age of onset is usually in the 4th decade, but is highly variable.

The pathological impact in SCA 17 is predominantly on the cerebellar cortex, the cerebral cortex, (notably in this context the cingulate and parahippocampal gyri), the striatum, the ventrolateral thalamic nucleus, and the mediodorsal thalamic nucleus [17]. The subthalmic nucleus and the substantia nigra are more mildly affected, and the cholinergic basal forebrain nuclei are spared [17].

Radiologically, SCA 17 displays a pattern of atrophy that typically involves both the cerebellar and cerebral hemispheres, and sometimes mild brainstem atrophy [26].

Clinical features are very varied [93], and patients may initially present with chorea, parkinsonism, psychiatric features and dementia but without ataxia, particularly with smaller (CAG)_n_ expansions (e.g., 43–47). As such, its clinical presentation may overlap that of Huntington’s disease or DRPLA (vide infra). Larger repeats usually present in early to mid-life with ataxia plus psychiatric features and cognitive impairment, followed by dementia (in about 90% of cases), with various combinations of parkinsonism, chorea and/or dystonia and spasticity ensuing ibid [94,95,96,97,98]. Indeed, the clinical presentation varies considerably even within the same family [99]. It is relevant to note that the following studies have identified cognitive features or dementia based on limited clinical testing or have used outcome measures expected to be affected by visuomotor impairments, and therefore the results may be regarded as inconclusive. De Michelle et al. [95] observed 10 patients with SCA 17 from two Italian families with disease onset mostly in the 3rd to 4th decade, and a single case with prominent anticipation of onset at age three years progressing rapidly to death at 15 years. Progressive cerebellar ataxia with early and severe dementia and psychiatric features (depression, insomnia, delusions) were frequent in the first family, with cerebral and cerebellar atrophy evident on MRI. In the second family, psychiatric features (depression, personality changes, aggressiveness, poor personal hygiene, delusional thought, hallucinations and alcoholism) were present at onset, with early cognitive impairment observed clinically and followed by ataxia, rigidity and dystonic movement.

Bruni et al. [94] found prominent behavioural problems and reduced verbal fluency in 13 of 16 affected family members, which progressed to a frontotemporal dementia characterised by loss of insight and deficits in attention/planning, distractibility, and critical thinking/judgement on neuropsychological testing. Apraxia and agnosia were absent. Clinical progression was fairly slow (approximately 30 years) with cerebellar signs emerging followed by extrapyramidal signs, and myoclonus and epilepsy appearing later. A case report by Neilsen et al. [98] points out the diagnostic challenge of neurodegenerative disorders such as SCA 17 in their discussion of a male referred at 44 years with rapid progression of personality changes and cognitive impairment over 1½ years. There was rapid deterioration in motor abilities and movement coordination with fine postural tremor of the hands, reduced speed of finger tapping, hand diadochokinesia, lower limb ataxia, and mildly broad-based gait. He had an MMSE score of 14/30 with neuropsychological testing revealing moderate to severe impairment in executive function, episodic memory (although not severely amnestic as he recognised 10/12 pictures out of 30), anomia, alexia and agraphia, and with signs of visuospatial impairment, although visuomotor tasks were used. Neuropsychological measures were not described or named (apart from the MMSE) and it is unlikely that visuomotor limitations were actively avoided. MRI showed mildly atrophic cerebellar hemispheres and vermis, and FDG PET showed markedly reduced metabolism in the cerebellum. There was also slightly reduced metabolism in the left caudate nucleus. Family history revealed that his mother was diagnosed with multiple sclerosis aged 44 years and was wheelchair bound, his maternal uncle was diagnosed with Parkinson’s disease and his maternal grandfather reportedly died from Parkinson’s disease. Molecular genetic analyses revealed that this proband had SCA 17.

In 12 SCA 17 patients compared with demographically-matched controls, MRI voxel-based morphology (VBM) revealed motor dysfunction that was associated with grey matter atrophy mainly affecting the cerebellum and other motor networks, particularly the basal ganglia. Psychiatric scores were associated with grey matter degeneration in the frontal and temporal lobe, cuneus and cingulum. This study restricted cognitive testing to the MMSE, finding a significant correlation between the MMSE score and atrophy of the nucleus accumbens [97]. In summary, SCA 17 is generally characterised by dementia with frontal features, psychosis, and personality change which was estimated to be present in 42% of patients in one study [97] compared with 26% observed in SCAs 1, 2, 3, 6, 7 and 8, and 47% in Huntington’s disease [100]. It is clear that disruptions in cerebrocerebellar and frontostriatal circuitry, together with likely additional extracerebellar compromise, underpin this debilitating disease. Of note, these studies emphasise the need to undertake careful and comprehensive clinical and neuropsychological examination of patients presenting with psychiatric symptoms and dementia, to enable precise diagnosis leading to appropriate management and counselling.

### 2.8. Dentatorubral Pallidoluysian Atrophy (DRPLA)

Although not designated as a SCA, DRPLA is often included with the SCAs as it typically manifests with progressive ataxia, amongst other features. DRPLA is caused by a (CAG)_n_ repeat expansion of between 49 and 79, resulting in an expanded polyglutamine tract in the atrophin-1 protein [101,102]. As with most other exonic (CAG)_n_ repeat expansion disorders (SCAs 6 and 17 excepted), DRPLA shows prominent anticipation, especially on paternal transmission (SCA 8 excepted). DRPLA is common in Japan, being responsible for about 10% of dominant ataxias. It is reported world-wide, although it is distinctly rare in Caucasians.

As its name implies, DRPLA results in atrophy of the dentate nucleus, the red nucleus, the pallidum and the subthalamic nucleus [103]. There is also considerable atrophy of the pontine and medullary tegmentum. Juvenile-onset patients tend to display generalised atrophy of cerebrum, brainstem and cerebellum, while patients with onset in adulthood and long disease duration may show white matter gliosis and hypomyelination [103]. The substantia nigra is spared. As with a number of other expanded polyglutamine tract disorders, intranuclear polyglutamine-containing inclusions are more widespread, but their relationship to neuronal dysfunction remains contentious [104].

MRI changes in DRPLA typically include brainstem and cerebellar atrophy. Cerebral atrophy may be seen in juvenile onset patients, and some adult patients with long disease duration show cerebral white matter T_2_ hyperintensity [105], correlating with the neuropathological changes noted above.

Clinically, ataxia and (at least after the first few years), cognitive decline are constants. Patients with onset before 20 years of age typically manifest myoclonus and epilepsy as well, while those with adult onset tend to manifest chorea and psychiatric disturbances in addition to ataxia and dementia [105].

While psychiatric features and dementia in patients with DRPLA have been reported in the literature, details of cognitive decline are relatively sparse and there is a scarcity of reports of comprehensive, or indeed, appropriate, neuropsychological assessment in these patients. Features of juvenile-onset DRPLA have included early social withdrawal, regression in behaviour, violent behaviour, increasing paucity of speech, mild intellectual disability, psychiatric diagnoses including autism and attention-deficit-hyperactivity disorder, and development of seizures [106]. Sunami et al. [107] described a father and son with typical presentations of adult-onset DRPLA and juvenile onset DRPLA, respectively. In this pedigree, the patient with adult-onset DRPLA demonstrated ataxia, chorea and dementia in middle-age, whereas his son with juvenile onset DRPLA mainly manifested ataxia, epilepsy, myoclonus and severe, young-onset dementia. MRI of the father’s brain showed atrophy of the cerebellum, midbrain and pons with dilation of the fourth ventricle, with more white matter involvement in the father than in the son. The son’s MRI, obtained at 35 years of age, showed more marked atrophy of the pons, cerebellum and both cerebral hemispheres than his father. A follow-up MRI at 39 years revealed marked progression of atrophic changes to the cerebellum, brain stem and both cerebral hemispheres. A similar pattern of DRPLA was observed in three generations of males in an Australian Macedonian family with DRPLA manifesting as very mild in elderly onset, moderate in young adult onset, and severe in childhood onset [108]. Cognitive decline in the childhood onset was characterised by learning difficulties observed at five years and increasing into primary school years, progressive myoclonic epilepsy at 13 years, and loss of vocabulary and basic language skills with marked deterioration in memory in teen years. His father’s cognition was grossly intact with the exception of reduced visual memory as measured on an unnamed task requiring memory for complex block constructions, and the grandfather was asymptomatic, albeit with mild ataxic features on examination. Cognitive testing reportedly administered to the latter consisted of motor tasks including the Color Trails test, the Rey Complex Figure copying task, and Luria tests for frontal lobe function [108]. Cognitive decline in adult onset DRPLA is reported in the literature to have a subcortical dementia profile, and psychosis may occur [109,110].

## 3. Conclusions

Cognitive changes are very frequent in the polyglutamine ataxias, but are often not apparent because appropriate psychometric instruments are rarely used for their assessment. Furthermore, the lack of consistency in choice of psychometric instruments, coupled with the paucity of studies directly comparing adequate numbers of subjects with different polyglutamine ataxias, followed longitudinally, makes comparison of their respective patterns and severities of cognitive impairment difficult. We tentatively suggest, however, that, based on neuropathological, radiological and clinical differences, as well as such information as is available from cognitive studies, these disorders might be classified into three groups. Firstly, there are those manifesting the CCAS only (SCAs 6 and 8, considered to be relatively “pure” ataxias). Secondly, there are those with more extensive deficits (but usually not actual dementia), consequent on involvement of structures beyond the cerebro-ponto-cerebello-thalamo-cortical loops (particularly the corticostriatal circuitry, and perhaps direct cortical involvement and/or cortical projections from the cholinergic basal forebrain nuclei). This group includes SCAs 1, 2, 3, and 7. (A caveat here is that some pedigrees with SCA 2 seem to have a high rate of dementia, although most do not.) The third group consists of SCA 17 and DRPLA, in which actual dementia with its consequent impairment of instrumental activities of daily living, is regularly encountered. However, despite the range in cognitive profiles and their severity, there are more similarities than there are clear-cut differences (with the possible exception of SCA 17 and DRPLA). Although patterns of cognitive impairment are therefore not particularly useful for clinical differential diagnosis amongst these disorders (again excepting SCA 17 and DRPLA), it is important to appreciate their existence, and that of the psychometric instruments that may be validly employed for their detection, when planning care for patients with these disorders.
